# Spatial Distribution and Hierarchical Clustering of β-Amyloid and Glucose Metabolism in Alzheimer’s Disease

**DOI:** 10.3389/fnagi.2022.788567

**Published:** 2022-06-06

**Authors:** Da-An Zhou, Kai Xu, Xiaobin Zhao, Qian Chen, Feng Sang, Di Fan, Li Su, Zhanjun Zhang, Lin Ai, Yaojing Chen

**Affiliations:** ^1^Department of Rehabilitation, The Third Affiliated Hospital of Jinzhou Medical University, Jinzhou, China; ^2^School of Artificial Intelligence, Beijing Normal University, Beijing, China; ^3^Department of Nuclear Medicine, Beijing Tiantan Hospital, Capital Medical University, Beijing, China; ^4^State Key Laboratory of Cognitive Neuroscience and Learning, Beijing Normal University, Beijing, China; ^5^Department of Psychiatry, University of Cambridge, Cambridge, United Kingdom

**Keywords:** Alzheimer’s Disease, glucose metabolism, hierarchical organization, spatial distribution, β-amyloid

## Abstract

Increased amyloid burden and decreased glucose metabolism are important characteristics of Alzheimer’s disease (AD), but their spatial distribution and hierarchical clustering organization are still poorly understood. In this study, we explored the distribution and clustering organization of amyloid and glucose metabolism based on ^18^F-florbetapir and ^18^F-fluorodeoxyglucose PET data from 68 AD patients and 20 cognitively normal individuals. We found that: (i) cortical regions with highest florbetapir binding were the regions with high glucose metabolism; (ii) the percentage changes of amyloid deposition were greatest in the frontal and temporal areas, and the hypometabolism was greatest in the parietal and temporal areas; (iii) brain areas can be divided into three hierarchical clusters by amyloid and into five clusters by metabolism using a hierarchical clustering approach, indicating that adjacent regions are more likely to be grouped into one sub-network; and (iv) there was a significant positive correlation in any pair of amyloid-amyloid and metabolism-metabolism sub-networks, and a significant negative correlation in amyloid-metabolism sub-networks. This may suggest that the influence forms and brain regions of AD on different pathological markers may not be synchronous, but they are closely related.

## Introduction

Alzheimer’s disease (AD) is a progressive neurodegenerative disease that usually has a slow progression and long course. The typical pathological feature of AD is extracellular β-amyloid protein (Aβ) deposition, which starts a decade or more before the onset of illness and appears to be a trigger of the pathological cascade of events leading to AD dementia. Observations suggest that Aβ deposition has reached a peak 10–12 years before the onset of AD symptoms, it is hypothesized that Aβ initiates tangle formation and neuronal cell death (Hardy and Allsop, 1991; Klunk et al., 2006). Recently, biomarkers have been emphasized in the diagnosis of AD. The National Institute on Aging and Alzheimer’s Association (NIA-AA) Research Framework state that Aβ changes, pathologic tau, and neurodegeneration (ATN) comprise the diagnostic standard of AD and highlight the importance of neuroimaging and fluid biomarkers for the accurate diagnosis of AD (Jack et al., 2018).

β-amyloid deposition accumulates early as disease progresses, and varies among brain regions, including deposition in some key regions which mediate cognition (Grimmer et al., 2009). The brain regions susceptible to Aβ accumulation comprise large areas of the medial and lateral association cortex in amyloid-positive individuals without dementia (Palmqvist et al., 2017). The posterior cingulate and the frontal and parietal cortices are most commonly regions affected early in AD and in mild cognitive impairment (MCI) due to AD (Kemppainen et al., 2006, 2007), which is consistent with other studies including post-mortem evaluations (Klunk et al., 2004; Driscoll et al., 2012). The presence of Aβ deposition in different brain regions at different stages may be associated with inconsistencies in the effects of Aβ on spatial areas of the brain, which may reflect regional differences in susceptibility to AD pathology. More recently, Aβ deposition in the cerebral cortex has been shown to have a hierarchical organization in elderly cognitively normal individuals, with four Aβ clusters based on spatial features (Sepulcre et al., 2017). It is uncertain if this hierarchical clustering organization of cognitively normal elderly is present in symptomatic AD patients and whether it reflects the spatial distribution of AD pathological changes.

β-amyloid is a critical hallmark in AD diagnosis whereas ^18^F-fluorodeoxyglucose (^18^F-FDG) positron emission tomography (PET) is a strong predictor of progression from MCI to AD dementia (Landau et al., 2010). In AD dementia patients Aβ deposition is widespread but is present in many individual who have not cognitive symptoms and it has a weak association with cognitive decline (Klunk et al., 2004). Reduced glucose metabolism is used as an indicator of synaptic dysfunction and neurodegeneration caused by Aβ. Patients with AD typically show temporal and parietal hypometabolism on FDG PET imaging in patients with AD (Ossenkoppele et al., 2012), where gray matter atrophy is common. Some studies have attempted to correlate metabolic function with the presence of Aβ deposition. However, the spatial distribution of hypometabolism and Aβ deposition is different in both AD patients and normal older adults (La Joie et al., 2012). A few multimodal imaging studies using FDG-PET and amyloid PET approached the question of whether local amyloid plaque deposition is correlated with local levels of glucose metabolism. These studies showed that the correlation was discordant, and changed with disease stages (Li et al., 2008; Cohen et al., 2009; Altmann et al., 2015). The spatial distribution relationship between the Aβ deposition and metabolism in AD, if any, remains uncertain. Studies have shown that Aβ tends to be deposited in core brain regions with higher structural and functional connections (Daianu et al., 2015) which may also be areas with high glucose metabolism.

The purpose of the present study was to examine the spatial distribution and extent of Aβ deposits and glucose metabolism and verify whether regions with high Aβ deposition are regions with high glucose metabolism by using florbetapir (^18^F-AV-45) and FDG PET. Additionally, we attempted to characterize a hierarchical structure of amyloid burden and metabolism organization that contains meaningful information about regional covariance patterns in AD patients. We further explored the relationship between regional Aβ deposition and glucose metabolism in AD patients.

## Materials and Methods

### Participants

Participants were selected from the Beijing Aging Brain Rejuvenation Initiative (BABRI) study, an ongoing longitudinal study examining the brain and cognitive decline in an elderly, community-dwelling sample (Li et al., 2013). All enrolled participants were Han Chinese, right-handed. Sixty-eight patients with AD dementia and 20 cognitive normal controls were included in the current study. All participants received a standard dementia screening that included medical history, physical and neurological examinations, brain CT or MRI and neuropsychological testing. All the AD patients were firstly diagnosed with AD when they were screened for cognitive problems from the BABRI cohort and were later referred to Beijing Tiantan Hospital, Capital Medical University. All enrolled participants (1) had no history of coronary disease, nephritis, tumors, neurological or psychiatric disorders, or addiction; (2) had no conditions known to affect cerebral function, including alcoholism, current depression, Parkinson’s disease, or epilepsy; and (3) had no large vessel diseases such as cortical or subcortical infarcts or watershed infarcts. Dementia was diagnosed based on criteria modified from DSM-5 and further evaluated by brain CT or MRI. The diagnosis of AD was made according to the criteria of the National Institute of Neurological and Communicative Disorders and Stroke and the Alzheimer’s Disease and Related Disorders Association (McKhann et al., 1984). Nine patients were CDR stage 0.5, 26 stage 1, 30 stage 2, and 3 stage 3. All patients were amyloid positive determined by visual read of florbetapir PET imaging by two experienced readers (XZ and LA). Control participants were amyloid negative determined by visual read of florbetapir PET scanning and denied any significant neuropsychiatric disease or memory trouble, were not taking any psychoactive medicines, and had a Mini Mental State Examination (MMSE) score of 26 or more and CDR = 0. The Ethics Committee and Institutional Review Board of Beijing Normal University approved this study (ICBIR_A_0041_002.02). For those AD patients who were unable to give informed consent, written, informed consent was obtained from their legal guardian.

### Positron Emission Tomography Image Acquisition and Data Analysis

All participants underwent a florbetapir PET scan and a ^18^F-FDG PET scan on a Discovery TM PET/CT Elite scanner (General Electric) at the Beijing Tiantan Hospital, Capital Medical University (Beijing, China). The florbetapir PET session that consisted of intravenous injection of 10 mCi of tracer followed by an uptake phase of 50 min. At 50 min patients were positioned in the scanner. FDG-PET scans were required to fast for 6 h before the injection of 185 ± 8 MBq of ^18^F-FDG. After approximately 60 min, an emission acquisition was performed. Native-slice thickness was 3.27 mm, with field of view 700/153. Florbetapir and FDG PET scans were acquired on different days, but within 1 week of each other. Florbetapir PET images were visually read by two experienced nuclear medicine physicians who were blind to the clinical data, and only Aβ-positive patients and Aβ-negative controls were included.

Positron emission tomography data were preprocessed using Statistical Parametric Mapping software version 12 (SPM12), and spatial normalization to Montreal Neurological Institute (MNI) templates was performed for all patients. We later analyzed the images using automatically detected regions of interest (ROI) from the LPBA40 template, an established set of 56 cortical and subcortical brain regions (LONI Probabilistic Brain Atlas, LPBA40) (Shattuck et al., 2008). Here, we analyzed all cortical regions (25 for each hemisphere) and calculated standard uptake value ratios (SUVRs) in each of the regions for both PET tracers, comparing them to the cerebellar gray reference.

### Statistical Analysis

Independent two-sample *t*-tests were used to assess between-group differences in age and MMSE score. The chi-square test was used to compare gender ratio difference.

(1) Percentage change calculation of Aβ deposition and glucose metabolism. AD have Aβ deposition and glucose hypometabolism in various brain regions, we used the relative change ratio of AD to normal controls to measure the degree of influence of AD on each brain region.


$$P\,e\,r\,c\,e\,n\,t\,a\,g\,e\,c\,h\,a\,n\,g\,e\,\left(i\right)=\frac{M_{\text{i}}\,\left(\text{AD}\right)-M_{\text{i}}\,\left(H\,C\right)}{M_{\text{i}}\,\left(\text{HC}\right)}\times 100$$

Here, *M*_i_(AD) is considered to be the mean SUVR of brain i for AD group, and *M*_i_(*HC*) means SUVR of brain i for controls.

(2) Hierarchical clustering analysis of brain amyloid load and metabolism. To determine whether the 50 cortical amyloid load or glucose metabolism can be classified into different categories, we performed the following hierarchical clustering analysis. The data vectors (florbetapir and FDG SUVR) for all regions used as input for cluster analysis. First, we treated each brain area as a cluster and calculated the Euclidean distance between every cluster pair, that is, the similarity between the brain areas. Next, we identified the two closest classes between the classes, grouped them together, and then recalculated the similarity between the generated class and the old classes. Finally, we repeated the above steps until all the clusters were grouped into one cluster together and the algorithm ended. When calculating the distance between clusters, the distance between the two sets of areas furthest from each other was taken as the distance between the two sets. In this way, we can divide all the brain areas into certain clusters by setting a certain distance after the algorithm is finished. The calculation process used the clustering function in MATLAB.

(3) Amyloid deposition and glucose metabolism correlations. For each Aβ or FDG hierarchical cluster, mean SUVR values were obtained by averaging the signals across all regions within each hierarchical clustering category. Pearson correlation coefficients between each pair of all Aβ and FDG categories were further computed to produce a symmetric correlation matrix for all patients, controlling for age, gender, and disease duration.

## Results

Characteristics of the study participants are given in Table 1. At the time of scan, patients with AD were on average 64.94 ± 8.14 years old. Forty-one percent of the patients were male and 88% had a Clinical Dementia Rating (CDR) score greater than one. There were no significant differences in chronic diseases like hypertension, type 2 diabetes mellitus and hyperlipidemia between these two groups.

**TABLE 1:** Sample characteristics.

Characteristics	Alzheimer’s disease (*n* = 68)	Healthy controls (*n* = 20)	*p*-value
Age (50–85 years)	64.94 ± 8.14	62.73 ± 9.62	0.301
Sex, M/F	28/40	9/11	0.801
CDR, 0/0.5/1/2/3	0/9/26/30/3	20/0/0/0/0	–
MMSE	12.31 ± 6.73	27.95 ± 1.36	<0.0001
AD duration (years)	2.69 ± 1.67	–	–
Hypertension, yes/no	15/53	4/16	0.844
Type 2 diabetes mellitus, yes/no	13/55	3/17	0.675
Hyperlipidemia, yes/no	18/50	7/13	0.457

*M, male; F, female; CDR, Clinical Dementia Rating; AD, Alzheimer’s disease; MMSE, Mini-Mental State Examination.*

### Amyloid Load and Glucose Metabolism Distribution in Alzheimer’s Disease

**FIGURE 1:**
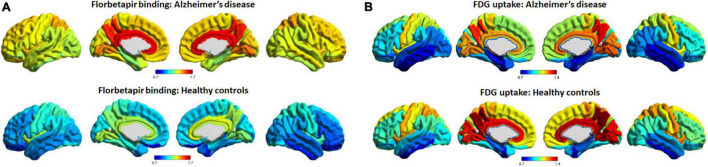
Average patterns of cortical florbetapir binding and FDG uptake (cerebellar reference). The spatial average patterns of cortical regional florbetapir binding **(A)** and FDG uptake **(B)** in AD patients and healthy controls. Color bars represent standard uptake value ratios. AD, Alzheimer’s disease; FDG, fluorodeoxyglucose.

**FIGURE 2:**
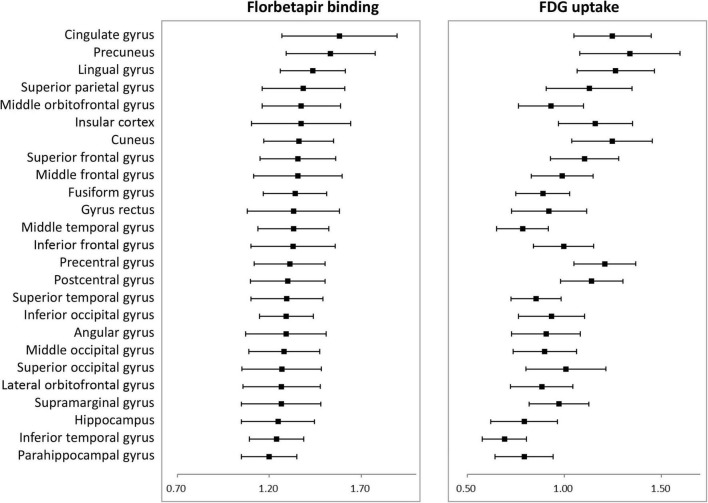
Average florbetapir binding and FDG uptake in 25 cortical areas in AD patients where error bars represent standard deviations. AD, Alzheimer’s disease; FDG, fluorodeoxyglucose.

Figure 1 shows average patterns of cortical florbetapir and FDG SUVR images of AD patients and cognitively normal elderly people. The highest amyloid load of regional florbetapir SUVR in AD patients was in cingulate gyrus, precuneus, lingual gyrus, followed by parietal and frontal areas, then by occipital and temporal regions. Many areas with high amyloid deposition are also areas with high glucose metabolism in AD patients, such as the cingulate gyrus, precuneus, lingual gyrus (Figure 2). To verify that areas with high amyloid load and metabolism in AD patients are indeed high and not unique to AD patients, we collected florbetapir and FDG-PET data from 20 cognitively normal elderly people. The results showed that both AD patients and normal elderly people had similar high and low metabolic consumption regions, such as the cingulate gyrus, precuneus, lingual gyrus, and cuneus with high glucose metabolism, while the hippocampal, parahippocampal gyrus, and inferior temporal gyrus had low glucose metabolism (Figure 1). Areas with high amyloid deposition are confirmed to be regions of the brain with high metabolic activity.

**FIGURE 3:**
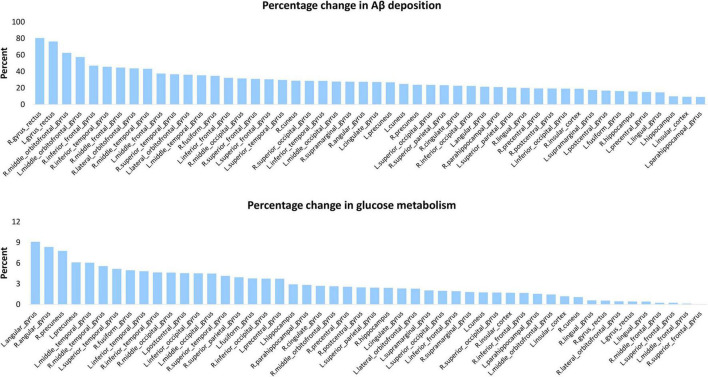
Percentage change of Aβ deposition and glucose metabolism for each cortical area. This chart displays the percentage increase of Aβ deposition (top panel) percentage decrease and of glucose metabolism (bottom panel) of AD vs. controls in 50 cortical areas.

β-amyloid deposition and glucose hypometabolism gradually spread to various areas of the brain in AD, and we calculated the percentage change to determine which areas were affected more severely in AD. Amyloid deposition in all cortical regions was significant higher in patients than in controls and percentage changes were highest in frontal and temporal lobes, with many areas exceeding 30%. Although the hippocampus and parahippocampal gyrus are early accumulated, the frontal and other temporal regions have a greater Aβ accumulation for the entire AD process. Metabolism was significant lower in patients in most areas, especially the parietal and temporal areas. For example, angular gyrus and precuneus are the areas with highest rate of change in patients, i.e., the areas with the most severe metabolic decline (Figure 3).

### Hierarchical Clustering of Cortical β-Amyloid Deposition and Glucose Metabolism in Alzheimer’s Disease

**FIGURE 4:**
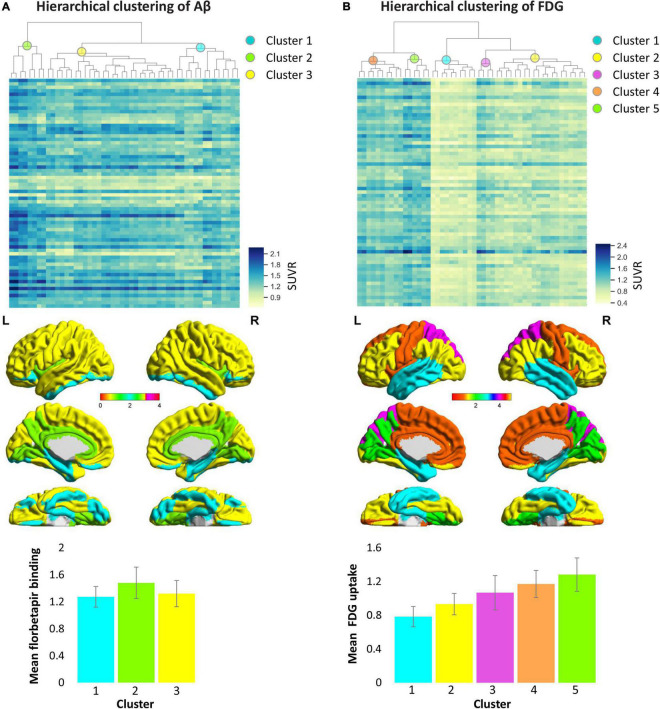
Hierarchical clustering of cortical Aβ deposition **(A)** and glucose metabolism **(B)** patterns in AD patients. Columns of heat maps correspond to the Aβ and FDG values of each cortical area, and rows correspond to samples. Color bars marked on the right indicates SUVR value, blue, highest; yellow, lowest. The dendrograms on the top show the classification results, which indicate the three categories from Aβ data and five categories from FDG data represented by the different colored spheres. The bar chart below shows the average SUVR values of each category in patients with Alzheimer’s disease. The colors in the bar chart correspond to categories in the brain maps. AD, Alzheimer’s disease; FDG, fluorodeoxyglucose; SUVR, standardized uptake value ratio.

Hierarchical clustering was used to construct the clusters of the brain amyloid load based on the regional Aβ and FDG data in AD patients. As shown in Figure 4, we set the distance to 2.2 and divided the all areas into three clusters for Aβ deposition and five clusters for metabolism. Among the three categories of Aβ deposition, category 1 mainly included the medial temporal lobe regions such as hippocampus and parahippocampal, category 2 mainly included the cingulate gyrus and precuneus, and category 3 included a wide range of cortical regions (Supplementary Table 1). The average deposition of amyloid in the three categories was calculated, and it was found that the deposition of category 1 was the lowest and that of category 2 was the highest (Figure 4A). Unlike the Aβ categories, the five categories of FDG showed more regionalization, where adjacent brain regions were clustered into one category. Among the five categories of FDG, category 1 mainly included the temporal lobe region, category 2 mainly included the lateral frontal, parietal lobe and occipital regions, category 3 mainly included the superior parietal gyrus and superior occipital gyrus, category 4 mainly included the cingulate gyrus, superior frontal gyrus, precentral and postcentral gyrus, and category 5 mainly included the precuneus, cuneus and lingual gyrus (Supplementary Table 1). The average glucose metabolism increased from category 1 to category 5 (Figure 4B).

### Amyloid Deposition and Glucose Metabolism Correlations

**FIGURE 5:**
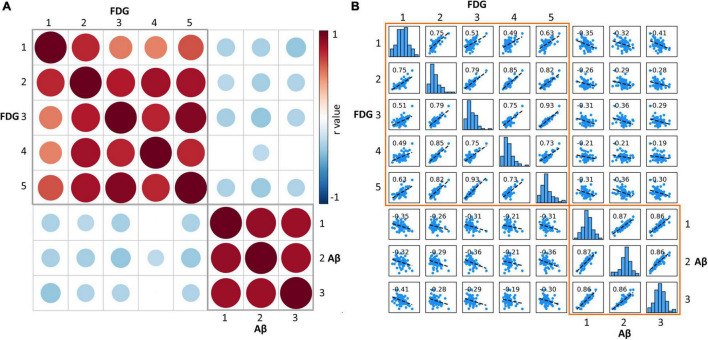
**(A)** Matrix of Pearson correlation coefficients between amyloid deposition and glucose metabolism for each category. The red circle represents significant positive correlation, the blue circle represents significant negative correlation, and the empty grid represents insignificant correlation. The size of the circle represents the size of the correlation coefficient. **(B)** Scatterplot matrix shows the correlation of each Aβ-FDG category pair. The diagonal-bar graph represents the SUVR value distribution of each category. FDG, fluorodeoxyglucose; SUVR, standardized uptake value ratio.

Correlation analysis (adjusted for gender, age, and disease duration) were used to assess the relationship between Aβ deposition and glucose metabolism in each hierarchical clustering category pair in patients with AD. Metabolism was significantly positively correlated between any two of the five FDG categories; florbetapir burden was significantly positively correlated between any two of the three Aβ categories. The correlation analysis between Aβ and FDG categories showed that the florbetapir burden of each Aβ category was negatively correlated with the metabolism of multiple FDG categories, that is, the metabolism of multiple FDG categories decreased with the increase of Aβ (Figure 5). It should be noted here that the previously mentioned “amyloid load and glucose metabolism distribution in AD” results indicated that areas with high Aβ deposition tend to be areas with high metabolism, compared with areas with low Aβ deposition. In this part, amyloid-metabolism correlation measures the relationship between the change rules of the two, that is, the degree of dependency and affinity.

## Discussion

This study examined the spatial distribution and hierarchical structure of amyloid burden and metabolism organization in AD patients. Several clear findings about the relationship between Aβ and energy metabolism are presented here. First of all, from the spatial distribution view, cortical regions with highest florbetapir binding like cingulate gyrus, precuneus, lingual gyrus, frontal and parietal areas, were also the regions with high glucose metabolism. This is consistent with several previous studies which described the topographic patterns of AD, MCI and HC groups (Klunk et al., 2004; Kemppainen et al., 2007; Rowe et al., 2007). Previous studies have shown that the cingulate gyrus and precuneus are the hub regions for structural and functional brain networks, which are central in brain communication and neural integration (van den Heuvel and Sporns, 2013). Frequent and massive information operations require high energy consumption. The posterior cingulate, precuneus and retrosplenial cortices together show the highest level of glucose use of any area of the cerebral cortex in humans (Gusnard and Raichle, 2001). These hub regions carry a large burden in everyday cognitive activities, making themselves prime targets for toxic metabolites accumulation like Aβ.

In terms of the magnitude of the change in Aβ deposition and glucose hypometabolism, some very interesting phenomena were found. Briefly speaking, the brain regions that changed the most in florbetapir binding and FDG uptake were not those regions with the highest absolute levels in those indicators. The percentage changes of amyloid deposition were greatest in the gyrus rectus and middle orbitofrontal gyrus, and the hypometabolism was greatest in the angular gyrus. The indicators of cingulate gyrus and precuneus ranked top by absolute magnitude, but they did not change the most from HC to AD. This is not surprising because these brain regions have already existed high deposition in the early stage of the disease (shown in Figure 1), and as the deposition of Aβ has a platform, it will not continue to quickly accumulate after a certain amount of accumulation. As a result, the final variability of these regions between AD and HC might probably not be as big as we thought. In contrast, the major indicators for determining the progression from mild to severe disease phase will be frontal and other temporal regions.

From the perspective of regional clustering, although the number of clustering of the two indicators was different, with three categories in florbetapir binding and five categories in FDG uptake, they were not that distinct. For example, Aβ cluster 1 and FDG cluster 1 are basically overlapped, mainly including the temporal lobe region such as hippocampus and parahippocampal, Pearson correlation coefficients between amyloid deposition and glucose metabolism of these two clusters was significant (*r* = −0.35). Another example, FDG cluster 5 is totally part of Aβ cluster 2 in anatomical location (*r* = −0.36). The similarity of location distribution and subsequent correlation analysis of different data clustering implies the internal relationship between these two indicators. Further, we also found Aβ deposit exerts a negative influence on energy metabolism not only in local areas, but also contralaterally distant brain areas. The underlying reason may include the following aspects. Aβ deposition appears to follow distinct pathways, spreading progressively through interconnected brain regions, rather than emerging from stochastic aggregation of Aβ in different brain areas over time (Heilbronner et al., 2013; Eisele and Duyckaerts, 2016; Condello and Stöhr, 2017). The distant brain areas with hypometabolism may be affected by propagated Aβ, the majority of which may be monomers or oligomers of Aβ. Oligmeric Aβ is reported to exert more toxic effects on neurons than fibrillar Aβ (Sun et al., 2015).

Decades before the onset of AD dementia, abnormal accumulation of insoluble amyloid proteins are detectable in the temporal lobe and association cortex (Villain et al., 2012; Grothe et al., 2017). It has been shown in vivo that Aβ deposits follow some degree of spatial specificity. In our study, we tried to describe the hierarchical spatial organization of Aβ pathology. With our hierarchical clustering analysis of amyloid PET data, we identified that the pattern of distribution of Aβ deposition in AD patients resembled the proposed Braak stages (Braak and Braak, 1991). The brain regions in the first cluster was closely linked with the amyloid pathology at the early stage, with areas of major changes being the fusiform, hippocampus, parahippocampal, rectus, lateral orbitofrontal, inferior temporal, and inferior occipital areas. The second cluster had almost closed spatial distribution with the amyloid pathology at the mid-stage, including cingulate gyrus, insular, lingual gyrus, and precuneus. The third cluster contained the majority of cortical areas, which correspond with amyloid pathology at late stage. The clustering results may suggest that some brain areas share similar pathological mechanisms, so that these areas are threatened by disease at the same stage.

This study helps us to comprehensively examine the pathological mechanism of AD from A multi-dimensional perspective, and researches about the pathological mechanism of Aβ from the perspective of energy metabolism are still not sufficient. Only a few multimodal imaging studies using FDG-PET and amyloid-PET approached the question of whether local amyloid plaque deposition is correlated with local levels of glucose metabolism. These studies showed that the correlation could be complex and changed with disease stages (Landau et al., 2012; Altmann et al., 2015). Some suggested that the amyloid deposition in MCI patients is associated with higher metabolism as a compensatory response (Cohen et al., 2009; Oh et al., 2014). However, negative correlations were observed between amyloid deposition and metabolism in AD patients (Landau et al., 2012; Grothe and Teipel, 2016), which is consistent with our observations. A mechanistic view linking accumulation of Aβ to the hypometabolism, however, has been lacking so far. The possible explanation underlying the association between Aβ and glucose metabolism may include insulin resistance (Neth and Craft, 2017; Kellar and Craft, 2020), mitochondrial dysfunction (involving TCA cycle and oxidative phosphorylation system), reactive oxygen species, apoptosis, inflammatory factors, excitotoxicity, glycation end products, hyper-activation of some protein kinases and so on (Devi et al., 2006; Chen and Zhong, 2013). Accumulating evidence suggests that mitochondrial dysfunction may play a fundamental role among these above pathways. Several *in vitro* studies posit that neurodegenerative disorders are associated with changes in mitochondrial dynamics and can be induced by Aβ that progressively accumulates within this organelle, acting as a direct toxin (Ferreira et al., 2010). Accumulation of the Aβ precursor protein, at mitochondrial membrane can cause mitochondrial dysfunction by blocking the translocation of other intra-mitochondrial molecules/proteins and disrupting the electron-transport chain (Sun et al., 2015). The Aβ localized in mitochondria can bind to two pro-apoptotic factors including Aβ-binding alcohol dehydrogenase and cyclophilin D, consequently increasing neurodegenerative cell death (Lustbader et al., 2004; Moura et al., 2010). Aβ induces activation of glutamate N-methyl-D-aspartate receptors and/or excessive release of calcium from endoplasmic reticulum that may underlie mitochondrial calcium dyshomeostasis thereby disturbing organelle functioning like energy conversion, and ultimately, damaging neurons (Ferreira et al., 2010).

There are limitations of our study. First, it is very important in the future to validate continuity and change in the AD progression by longitudinal studies in cohorts including MCI. Interrogation of a longitudinal dataset is also warranted to verify the hierarchical clustering results from our cross-sectional analyses. AD in this sample has likely been present longer than recorded, given the difficulty of identifying and documenting early cognitive changes. It remains unclear if apolipoprotein E gene is implicated in the AD-related effects of Aβ load patterns and this should be addressed in future studies.

In summary, we demonstrated that cortical regions with more Aβ accumulation were the regions with high glucose metabolism. The hierarchical clustering provides evidence that Aβ accumulation and glucose metabolism are region-specific and regions in the same cluster may be specifically affected in AD. Amyloid in each hierarchical category is significantly negatively correlated with metabolism in multiple categories supporting the hypothesis that Aβ deposition is an early event of the pathological process and relates to neurodegenerative changes of multiple brain regions.

## Data Availability Statement

The raw data supporting the conclusions of this article will be made available by the authors, without undue reservation.

## Ethics Statement

The studies involving human participants were reviewed and approved by the Ethics Committee and Institutional Review Board of Beijing Normal University. The patients/participants provided their written informed consent to participate in this study.

## Author Contributions

YC had full access to all of the data in the study and took responsibility for the integrity of the data and accuracy of the data analysis. YC and LA conceived the original idea for the study, supervised the conception, and revised and drafted the manuscript. YC, D-AZ, KX, XZ, QC, FS, DF, ZZ, and LA recruited the study population and conducted the neuropsychological tests. YC, FS, and LS analyzed the data. All authors read and approved the final manuscript.

## Conflict of Interest

The authors declare that the research was conducted in the absence of any commercial or financial relationships that could be construed as a potential conflict of interest.

## Publisher’s Note

All claims expressed in this article are solely those of the authors and do not necessarily represent those of their affiliated organizations, or those of the publisher, the editors and the reviewers. Any product that may be evaluated in this article, or claim that may be made by its manufacturer, is not guaranteed or endorsed by the publisher.
