# Supplementary material for: Spatial Distribution and Hierarchical Clustering of β-Amyloid and Glucose Metabolism in Alzheimer’s Disease
Source: Front Aging Neurosci. 2022 Jun 6;14:788567. doi: 10.3389/fnagi.2022.788567 (PMC9207533; doi:10.3389/fnagi.2022.788567)
Supplement: Supplementary file 1 [file Table_1.DOCX]

**Table S1. Regional FDG uptake in patients with Alzheimer’s disease and healthy controls.**

| Brain area | Aβ hierarchical cluster | FDG hierarchical cluster |
| --- | --- | --- |
| Fusiform gyrus | 1 | 2 |
| Gyrus rectus | 1 | 2 |
| Hippocampus | 1 | 1 |
| Inferior occipital gyrus | 1 | 2 |
| Inferior temporal gyrus | 1 | 1 |
| Lateral orbitofrontal gyrus | 1 | 2 |
| Parahippocampal gyrus | 1 | 1 |
| Cingulate gyrus | 2 | 4 |
| Insular cortex | 2 | 4 |
| Lingual gyrus | 2 | 5 |
| Precuneus | 2 | 5 |
| Angular gyrus | 3 | 2 |
| Cuneus | 3 | 5 |
| Inferior frontal gyrus | 3 | 2 |
| Middle frontal gyrus | 3 | 2 |
| Middle occipital gyrus | 3 | 2 |
| Middle orbitofrontal gyrus | 3 | 2 |
| Middle temporal gyrus | 3 | 1 |
| Postcentral gyrus | 3 | 4 |
| Precentral gyrus | 3 | 4 |
| Superior frontal gyrus | 3 | 4 |
| Superior occipital gyrus | 3 | 3 |
| Superior parietal gyrus | 3 | 3 |
| Superior temporal gyrus | 3 | 1 |
| Supramarginal gyrus | 3 | 2 |
